# Gray Matter Alterations Associated With Dissociation in Female Survivors of Childhood Trauma

**DOI:** 10.3389/fpsyg.2019.00738

**Published:** 2019-04-05

**Authors:** Judith K. Daniels, Anna Schulz, Julia Schellong, Pengfei Han, Fabian Rottstädt, Kersten Diers, Kerstin Weidner, Ilona Croy

**Affiliations:** ^1^Division of Clinical Psychology and Experimental Psychopathology, Department of Psychology, University of Groningen, Groningen, Netherlands; ^2^Psychologische Hochschule Berlin, Berlin, Germany; ^3^Abteilung für Psychotherapie und Psychosomatik, Medizinische Fakultät Dresden, Technische Universität Dresden, Dresden, Germany; ^4^Abteilung für Psychologie, Technische Universität Dresden, Dresden, Germany; ^5^Abteilung für Hals-Nasen-Ohren-Heilkunde, Medizinische Fakultät Dresden, Technische Universität Dresden, Dresden, Germany

**Keywords:** childhood trauma, dissociation, VBM, gray matter, temporoparietal junction, posttraumatic stress disorder

## Abstract

**Objective:**

Across various axis-1 disorders, the severity of dissociative symptoms is significantly related to a history of childhood traumatization. Thus, the question arises if coping with childhood trauma leads to neural adaptations that enhance the frequency of dissociative processing during adulthood. The aim of the two reported studies therefore was to identify and replicate gray matter alterations associated with dissociation.

**Methods and Results:**

In a first study, whole-brain MRI data were acquired for 22 female in-patients with trauma-spectrum disorders and a history of severe childhood trauma. Voxel-based morphometry (VBM) was carried out to test for significant correlations between dissociation (depersonalization/derealization) severity and gray matter volume. Dissociation severity was positively associated with volume in the left angular gyrus. This result was diagnosis-invariant. The replication study involved 26 female in-patients with trauma-spectrum disorders and 25 healthy controls. No significant association between dissociation severity and brain volume in a left angular gyrus region of interest located at the peak identified in study 1 was identified and no significant group difference in this region could be established.

**Conclusion:**

The angular gyrus has previously been implicated in the processing of agency and vestibular integration as well as dissociative processing. The current attempt at a direct replication of brain volume alterations however, failed. The data thus only partially support the notion that dissociative processing is associated *trans*-diagnostically with structural brain alterations in the left angular gyrus and independent replication in a larger patient sample is essential.

## Introduction

Over the course of the last years, it has become increasingly clear that pathological levels of dissociative symptoms occur not only in patients suffering from dissociative disorders, but also in a variety of axis-I disorders (Lyssenko et al., 2018). Recently, several authors have therefore called to study dissociation as a *trans*-diagnostic process rather than a disorder-specific alteration (Sar, 2014). These disorders have in common that patients afflicted by them often report a history of childhood trauma and that the severity of the dissociative symptoms is often significantly correlated with the severity of childhood trauma reported (Lochner et al., 2004; Bob et al., 2005; Molina-Serrano et al., 2008; Imperatori et al., 2015). Childhood abuse is also a risk factor for posttraumatic stress disorder (PTSD). Accordingly, dissociative symptoms are regularly reported by patients suffering from PTSD (Hopper et al., 2007; Daniels et al., 2012; Steuwe et al., 2012) and up to 25% of patients with PTSD following childhood abuse can be classified as belonging to the dissociative subtype (Steuwe et al., 2012; Wolf et al., 2012). Dissociation in PTSD as well as various patient samples typically involves transient episodes of depersonalization and derealization, but not identity confusion or amnesia for current events.

We hypothesize that dissociative coping mechanisms in response to childhood maltreatment are associated with structural alterations – independent of the development of specific axis-1 disorders. Such anatomical differences may occur either via plastic adaptation to function or as a preexisting condition fostering function. Analyzing those structural alterations might help to further elucidate the neural correlates of dissociative processing.

Research on morphological alterations related to depersonalization and derealization is, however, still rare and focused mainly on patients with PTSD. Dissociative symptoms in PTSD patients are positively related to enhanced volume of the medial superior frontal gyrus (Nardo et al., 2012; Daniels et al., 2016) and the bilateral temporal poles (Nardo et al., 2012), both playing an important role in the cognitive control network, as well as in the angular gyrus – an area implicated in the processing of agency and vestibular integration. Convergently, electric stimulation of the right angular gyrus induced out-of-body phenomena in a patient with epilepsy (Blanke et al., 2002).

Another line of research focused on depersonalization/derealization disorder (DPD). Within the dissociative disorders, DPD is the most alike to the set of dissociative symptoms reported by patients suffering from other axis-1 disorders, the difference being that in DPD the symptoms are typically chronic, often present without any fluctuation, and include excessive emotional numbing. DPD is a highly disabling condition and constitutes a dissociative disorder in its own rights. Several large-scale investigations of DPD indicate that this disorder is not consistently associated with severe childhood trauma (Baker et al., 2003; Daniels et al., 2015; Michal et al., 2016). The etiology as well as the neural correlates might therefore differ significantly from those of trauma-related disorders with dissociative symptoms. Two structural MRI studies using different methodological approaches have been published to date, which did not show any overlap of neural correlates with dissociation-associated alterations reported in PTSD samples. Reduced gray matter volume in the right caudate, right thalamus, and right cuneus as well as volume increases in the left dorsomedial prefrontal cortex and right somatosensory region has been reported in DPD as compared to healthy controls (Daniels et al., 2015). With regard to cortical thickness, a second investigation evidenced significant reductions in DPD in bilateral temporal lobes, inferior frontal regions, the right posterior cingulate, and increased thickness in the right gyrus rectus and left precuneus (Sierra et al., 2014).

It is thus currently unknown whether dissociation-associated brain alterations can be identified *trans*-diagnostically in subjects with a history of childhood trauma. Morphological patient studies have previously been criticized for both reporting biases (Fusar-Poli et al., 2014) and limited reliability due to small clinical samples sizes (Button et al., 2013). To counteract these potential effects, we opted to conduct both an exploratory study and a confirmatory replication conjointly.

### Aims of the Study

In Study 1, we aimed to examine the association between trait dissociation and brain anatomy in patients with a history of childhood trauma across a naturally divergent set of trauma-related disorders. We conducted a whole-brain analysis but hypothesized based on previous publications that (a) dissociative processing following childhood trauma is associated with specific neural alterations in structures such as the angular gyrus, superior frontal region, and temporal poles, and (b) those alterations exist *trans*-diagnostically.

Study 2 was conducted in order to replicate the results of Study 1 using a confirmatory analysis limited to the brain structures identified in Study 1. In addition, the patient data were compared to healthy controls.

## Materials and Methods

### Participants

All patients were recruited from an in-patient treatment program at the Clinic of Psychotherapy and Psychosomatic Medicine of the University Hospital Dresden. The clinic is specialized in the treatment of patients with a history of severe childhood trauma who express symptoms of dissociation, emotion regulation difficulties, and PTSD – a disease pattern often described as complex PTSD. Clinical diagnostics as well as detailed anamnestic interviews were performed by trained psychotherapists in all patients. In addition, the Structured Clinical Interview for DSM-IV Diagnosis (SCID; First et al., 1996) was performed by trained examiners with each patient and supported clinical axis-1 diagnoses.

In order to delineate the neural correlates of dissociative symptomatology from potential effects of childhood maltreatment, only patients with a clear history of significant childhood trauma were included. Criterion was a score of ≥11 on the childhood trauma questionnaire, indicating a value within or above the 95th percentile in the American (Scher et al., 2001) and German normative samples (Iffland et al., 2013). Participants reporting childhood trauma in the anamnestic interview but not in the questionnaire, or vice versa, were not included in the study. To ensure ecological validity, we generally did not exclude patients with comorbid disorders, except for patients with psychotic disorders, bipolar disorder, or dissociative identity disorder. Further exclusion criteria were pregnancy, insufficient command of the German language, and implanted devices that are not MR safe. All patients volunteered for two previously published studies, in which the impact of mental health on olfactory processing was assessed using functional MRI (Croy et al., 2010, 2014

Both studies were conducted according to the Declaration of Helsinki and approved by the local ethical board of the Technical University of Dresden. After complete description of the study, written informed consent was obtained from all participants.

In Study 1, data from 22 female in-patients aged 22–59 years (mean age 39.6, SD 10.3 years, compare Supplementary Table S1 for more detailed information) were reanalyzed for the purpose of the study.

In this in-patient sample, comorbidity was the norm rather than the exception. On average, each patient was diagnosed with 3.4 mental disorders. In total, 64% of the patients suffered from major depression (F32, F33: *n* = 14) and 64% suffered from PTSD (F43.1: *n* = 14). Further, somatoform disorders (F45: *n* = 9), personality disorders (F60-61: *n* = 7), anxiety disorders (F40-41: *n* = 4), and a history of substance abuse (F1: *n* = 4) were frequent. 59% were diagnosed with other mental disorders (*n* = 13).

All but one patient were on medication, with the majority prescribed antidepressants (compare Supplementary Table S1). Patients were administered the questionnaires at inclusion for the study, i.e., on average after approx. 4 weeks of treatment.

The replication sample in Study 2 consisted of 26 women (aged 24–57 years, mean 41.5, SD 10.1 years; compare Supplementary Table S1) which were compared to 25 healthy women (aged 20–69 years, mean 39.7, SD 13.9 years). There were no age differences between patients recruited for Study 1 and Study 2 [t(46) = 0.6; *p* = 0.18].

Again, comorbidity was rather the norm than the exception and patients were diagnosed with 3.03 mental disorders on average. In this sample, 92% of the patients suffered from major depression (F32, F33: *n* = 24, compare Supplementary Table S1) and 92% suffered from PTSD (F43.1: *n* = 24). Furthermore, anxiety disorders (F40-41: *n* = 18), somatoform disorders (F45: *n* = 8), and personality disorders (F60-61: *n* = 2). 12% were diagnosed with other mental disorders (*n* = 3). Similar to Study 1, most patients were on medication, mainly antidepressants (compare Supplementary Table S1). In contrast to Study 1, patients were asked to fill in the assessment instruments upon admission to in-patient treatment.

The control group was matched for age [t(49) = 0.5; *p* = 0.60] and current mental health problems assessed with the Patient Health Questionnaire (Lowe et al., 2004) served as exclusion criteria.

### Assessment Instruments

All participants completed the childhood trauma questionnaire (CTQ; Bernstein et al., 1997; Wingenfeld et al., 2010) and the beck depression inventory (BDI-II; Hautzinger et al., 1995; Beck et al., 1996). Dissociation severity was assessed using the 44-item Fragebogen zu dissoziativen symptomen (FDS), which is based on the Dissociative Experiences Scale, but contains an additional 16 items (Carlson and Putnam, 1993; Spitzer et al., 1998). Each FDS item is formulated as a statement of dissociation and the patient is required to indicate for each item, how many percent of the time this occurs to him/her. A mean score is computed over the percentages to determine overall dissociation severity.

### MRI Acquisition

All imaging data of Study 1 were collected using a 1.5 T MRI scanner (Sonata, Siemens Medical Solutions, Erlangen, Germany) with the manufacturer’s 8-channel phased array head coil. T1-weighted anatomical images of the whole head with 1mm isotropic resolution using a 3D magnetization prepared gradient rapid acquisition gradient echo (MPRAGE) sequence (TR/TE/TI = 2180 ms/3.39 ms/1100 ms, sagittal orientation, FOV (X,Y,Z) = 352 mm × 352 mm × 384 mm; reconstructed voxel size: 1 mm^∗^1 mm^∗^1 mm). For Study 2, imaging data were collected on a 3 T Siemens Magnetom Verio scanner (Siemens Healthcare, Erlangen, Germany) with an 8-channel phased-array head coil. T1-weighted anatomical images of the whole head were acquired (MPRAGE sequence; TR/TE/TI = 2530 ms/2.34 ms/1100 ms, sagittal orientation, FOV 256^∗^256^∗^192 mm; voxel size 1 mm × 1 mm × 1 mm).

### VBM Processing

Separately for both studies, VBM analysis was performed using SPM12 (Wellcome Trust Centre for Neuroimaging, London, United Kingdom^1^) and Matlab R2015b (MathWorks, Natick, MA, United States) with settings recommended by Ashburner (2010). First, all T1-weighted anatomical images were manually re-oriented to place the anterior commissure at the origin of the three-dimensional Montreal Neurological Institute (MNI) space. Subsequently, images were automatically segmented into gray matter, white matter, and cerebrospinal fluid (CSF). The DARTEL (diffeomorphic anatomical registration through exponentiated Lie algebra) algorithm was used to generate a group-specific template. A flow field storing the deformation information for warping the participant’s scans onto the template was created for every participant. These were then used to spatially normalize gray matter images to MNI space employing affine spatial normalization as implemented in the normalization algorithm included in the DARTEL toolbox. This last step comprised modulation and smoothing with an 8-mm full-width half-maximum (FWHM) isotropic Gaussian kernel (Ashburner, 2010). Anatomical labeling was carried out using the anatomy toolbox (Eickhoff et al., 2005) embedded in SPM 12.

### Statistical Analyses

In Study 1, voxel-wise correlation of gray matter volume with dissociation severity (mean percentage score on the FDS) was performed using multiple regression analyses with absolute threshold masking at 0.2. Total gray matter volume (analyzed with the SPM extension Easy Volumes^2^) was used for global normalization to account for differences in brain size.

The three classes of covariates (childhood trauma severity, depression severity, and medication), were added as nuisance effects in separate covariance analyses.

As dissociation severity was significantly correlated with childhood trauma severity in Study 1, we also added the mean CTQ value of each participant as a covariate. In order to test whether the diverging results observed in the two samples can be fully attributed to differences in medication status or depression (BDI score), we *post hoc* added additional analyses controlling for these group differences. As different classes of medications are known to have different effects on brain activation (Lanius et al., 2010) and morphometry (Thomaes et al., 2014) in PTSD patients, the intake of each of five classes of medication (antidepressiva, neuroleptica, tranquilizer, anticonvulsants, and analgetics) was added per participant as a separate covariate for each sample.

For the MRI data, we opted to use a statistical threshold of *p* < 0.001 in combination with a non-stationary threshold to balance the risks of Type-I and Type-II errors (Lieberman and Cunningham, 2009) for all analyses. Non-stationary thresholding corrects for whole-brain comparisons by determining an additional minimum extent threshold for each analysis. The non-stationary extent threshold was computed with the VBM-8 toolbox for SPM^3^ to adjust for multiple comparisons at a significance level of *p* < 0.05. The remaining statistical analyses were conducted using SPSS^4^, applying a statistical significance threshold of *p* < 0.05 and *k* > 20.

In Study 2, we carried out confirmatory testing of the main result of Study 1 by extracting averaged VBM parameter estimation from an 8 mm sphere built around the angular gyrus peak obtained in Study 1 (-45 -56 24) for each individual. In addition, the data were analyzed for the group of patients and controls separately by replicating the exploratory whole-brain, voxel-wise correlation of gray matter volume with dissociation severity following procedures outlined above (using multiple regression analyses with absolute threshold masking at 0.2 and total gray matter volume used for global normalization) in order to ensure that our null-finding was not due to a slight misplacement of the sphere used for confirmatory testing. In order to test whether the diverging results in the two patient groups might be due to differences in sample composition, we *post hoc* added several covariates to test how stable the null-finding is. We accounted (1) for potential effects of medication by covarying the intake of each of the five types of medication (antidepressiva, neuroleptica, tranquillizer, anticonvulsants, and analgetics), (2) covaried the severity of CM, and (3) covaried depression severity by using those variables as regressors of no interest in the analysis.

In a separate analysis, the gray matter volume of patients and controls were contrasted against each other under control of the total gray matter volume. In order to unmask the effect of dissociation, the group comparison was done with and without inclusion of the FDS value as a regressor of no interest.

## Results

### Study 1 – Discovery Sample

#### Sample Characteristics

FDS scores assessing dissociation severity ranged from 3.2 to 61.4 (mean score of 24.7 ± 15.9 SD, first quartile below 9.3, the second below 26.9, and the third below 36.6) and depression scores on the BDI ranged from 8 to 51 (mean 26.5 ± 10.6 SD), and childhood trauma severity measured with the CTQ ranged from 11.2 to 22.4 (mean 15.5 ± 3.1 SD, see Supplementary Table S1). Severity of dissociation correlated significantly with severity of childhood trauma (*r* = 0.52, *p* = 0.014), but not with depression levels (*r* = 0.37, *p* = 0.09).

#### VBM Results

A significant positive correlation between dissociation severity and gray matter volume in the left angular gyrus, extending to the middle temporal gyrus was identified. A similar gray matter enhancement was observed on the contralateral side (MNI coordinate 48, -59, 26; *T* = 2.97, *p* = 0.004), but missed statistical significance. An additional cluster was observed in the left inferior frontal gyrus, pars triangularis, however this did not hold for non-stationary cluster extent correction and is only reported in order to get a comprehensive picture (see Table 1). There were no significant reductions of volume in relation to dissociation severity.

**Table 1:** Results of Study 1 – gray matter volume in relation to dissociation severity.

MNI coordinates			T score	Cluster size *k*	Brain region
**Positive correlation with dissociation severity (FDS scores) in whole sample (*n* = 22) at *p* < 0.001, in bold corrected for multiple comparisons**					
**at *k* > 129**					
**-45**	**-56**	**24**	**6.52**	**356**	**Left angular gyrus extending to left middle temporal cortex**
-39	42	3	3.94	35	Left inferior frontal gyrus (pars triangularis)


*Results significant after non-stationary cluster extent correction for multiple comparisons are printed bold. Non-stationary thresholding is used to correct for whole-brain comparisons. A specific extent threshold is determined per analysis. All other analyses were thresholded at *p* < 0.001 with a cluster extend of *k* = 20. No significant negative correlation between dissociation severity and brain volume was detected. *k*, cluster extent (in voxels); FDS, Fragebogen für dissoziative symptome.*

This effect was not solely driven by the subjective severity of the childhood trauma: After inclusion of the CTQ score as a covariate, the correlation between dissociation severity and gray matter enhancement in the left angular gyrus remained intact, but decreased in cluster width and strength (compare Supplementary Table S2). After covariation of depression severity, the left angular gyrus remained significantly associated with dissociation severity and after covariation of five medication classes, the left angular gyrus still showed a strong correlation, but missed the extent threshold (see Supplementary Table S2 for additional results).

#### Study 2 – Replication Sample

##### Sample characteristics

For patients, FDS scores assessing dissociation severity ranged from 5.5 to 79.0 (mean score of 31.8 ± 19.3 SD, first quartile below 20.1, the second below 26.5, and the third below 43.0), depression scores ranged from 23 to 62 (mean 39.2 ± 9.5 SD) and childhood trauma severity ranged from 11.4 to 24.6 (mean 17.5 ± 3.9 SD, compare Supplementary Table S1). Hence, patients from Study 2 did not differ significantly from those recruited for Study 1 in terms of dissociation severity [*t*(46) = 1.4; *p* = 0.18] and childhood maltreatment [*t*(46) = 1.9; *p* = 0.06], but they reported significantly stronger depression [*t*(46) = 4.4, *p* < 0.001].

In this sample of patients, severity of dissociation did not correlate significantly with severity of childhood trauma (*r* = 0.12, *p* = 0.58) or depression levels (*r* = 0.28, *p* = 0.16).

Controls reported the expected low values for dissociation (range 0–36; mean 5.7 ± 8.5 SD), depression (range 0–14, mean 4.4 ± 3.6 SD), and childhood maltreatment (range 0–9.2, mean 4.5 ± 2.9 SD). As compared to patients, controls did report significantly lower values for dissociation [*t*(49) = 6.2; *p* < 0.001, *d* = 2.5], depression [*t*(49) = 9.1; *p* < 0.001, *d* = 4.3], and childhood maltreatment [*t*(49) = 13.4; *p* < 0.001, *d* = 3.7]. Dissociation scores did not correlate significantly with childhood trauma (*r* = 0.01, *p* = 0.70) or with depression scores (*r* = 0.18, *p* = 0.49) in the control group. As dissociation scores deviated significantly from a normal distribution in controls, Spearman correlation was used for the latter statistics.

##### VBM results

**FIGURE 1:**
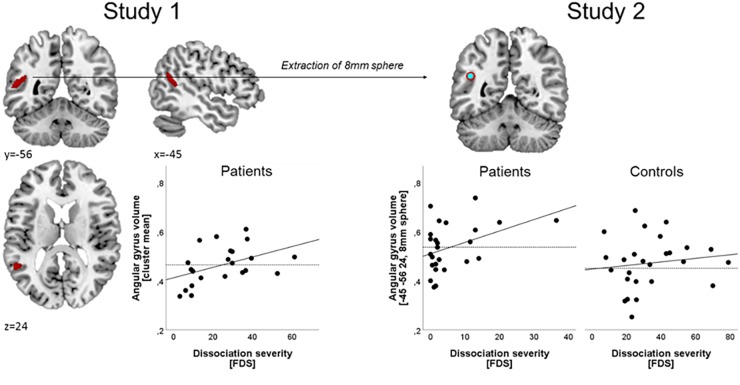
Relation between gray matter volume and dissociation severity. Left panel: in Study 1 (*N* = 22 female patients), a significant correlation was obtained between dissociation severity, and a cluster in the left angular gyrus (thresholded at *p* < 0.001 and *k* > 129). Dissociation severity was operationalized as the mean percentage on the *Fragebogen zu dissoziativen symptomen* (FDS). Right panel: In Study 2, a sphere of 8 mm was built around the angular gyrus peak voxel obtained from study one and VBM estimates in this ROI were participant-wise extracted in a group of *n* = 26 patients and *n* = 25 controls. Those estimates did not correlate significantly with dissociation severity in either group. No significant group differences were observed for the mean value (dashed line) between patients and controls.

The beta values extracted from the 8 mm sphere around the significant angular gyrus peak observed from Study 1 did neither correlate significantly with dissociation severity in the patient group (*r* = 0.13, *p* = 0.53; Figure 1) nor in the control group (*r* = 0.32, *p* = 0.12). To test whether the replication in the patient group failed due to the significantly higher depression scores, the correlation between dissociation severity and brain volume in the angular gyrus was repeated while covarying out depression scores. However, taking depression severity into account did not render this correlation significant (*r* = 0.12, *p* = 0.57, see Supplementary Table S3). Similarly, covariation for medication status and childhood trauma severity did not render the correlation significant (see Supplementary Table S3).

Compared to controls, patients exhibited a significantly reduced volume in the left (MNI coordinate: -35 6 60, *T* = 4.29, *k* = 231) and right middle frontal gyrus (MNI coordinate: 41 33 35, *T* = 5.87, *k* = 615) and in the thalamus (MNI coordinate: 6 -6 5, *T* = 4.32, *k* = 187) and no significant volume increases were observed in the group of patients. After inclusion of the FDS as covariate, the volumetric differences between groups were limited and only the left inferior frontal gyrus (MNI coordinate: -48 11 5, *T* = 4.03, *k* = 131) was found to be significantly reduced in the group of patients.

## Discussion

The main result of this investigation was that while a significant association between dissociation severity and gray matter volume was established *trans*-diagnostically in one sample, the replication attempt in a second patient sample failed. It is worth noting that both patient samples exhibit high average dissociation scores as compared to other clinical populations (Spitzer et al., 2015).

In Study 1, a significant positive correlation between dissociation severity and gray matter volume in the left angular gyrus, extending into the middle temporal lobe, was established in female patients with a history of severe childhood trauma *trans*-diagnostically. When severity of childhood trauma was covaried, the correlation between dissociation and gray matter volume of the left angular gyrus cluster (126 voxels) only marginally missed the extent threshold, indicating that it was mostly driven by the more proximal dissociation variable. Similarly, depression severity and medication status cannot fully account for this association, albeit the detected cluster were also smaller in size in these *post hoc* covariation analyses (depression covaried: 172 voxels; medication covaried: 56 voxels).

The angular gyri are involved in many different functions and are considered a cross-modal integration hub (for a comprehensive review see Seghier, 2013). As such, ischaemic lesions of the left angular gyrus have been associated with functional impairments across several modalities as well as autoscopia (Ripellino et al., 2013). In particular, they are thought to be involved in vestibular integration and the processing of agency (David et al., 2006; Farrer et al., 2008), with differences in individual hemispheric lateralization (Farrer et al., 2008). Agency, the feeling that one is initiating, executing, and controlling one’s own volitional actions, has been described as a key aspect of the bodily self and self-other discrimination (Farrer et al., 2008). A reduction in perceived agency is also a hallmark of dissociative processing, with patients reporting to feel like an observer of their own actions. The emergence of agency is currently mainly understood as the result of a complex feed-forward model, in which efference copies of motor actions are used to predict the sensory outcome of these actions (Friston, 2012; Seth et al., 2011). The fit between the two thus allows the body to differentiate between self-generated actions and external events. It has been suggested that a disruption of this intricate feed-forward system leads to dissociative symptoms – from reduced subjective agency to full-blown out-of-body sensations (Daniels, 2006; Gaebler et al., 2013). Convergently, neurological lesions of the bilateral angular gyri seem to be associated with severe dissociative symptoms and out-of-body phenomena (Bunning and Blanke, 2005; Brandt et al., 2005), while transcranial magnetic stimulation of the right angular gyrus impaired mental own-body transformation in a task developed to mirror out-of-body phenomena (Blanke et al., 2005). Dissociation severity was positively correlated with gray matter volume of the right angular gyrus in subjects with PTSD (Nardo et al., 2012) and with the right middle temporal lobe in borderline personality disorder with comorbid PTSD (Niedtfeld et al., 2013). In patients with dissociative identity disorder, which is commonly associated with extreme levels of childhood trauma (International Society for the Study of Trauma and Dissociation, 2011), a hyperactivation of the right angular gyrus has been reported (Reinders et al., 2014). In contrast, depersonalization/derealization disorder, which is not consistently associated with elevated levels of childhood trauma, has been associated with reduced energy metabolism as well as reduced volume of the right angular gyrus (Simeon et al., 2000; Daniels et al., 2015). There is also preliminary evidence that transcranial magnetic stimulation of the right angular gyrus can elicit a significant symptom reduction in some DPD patients (Jay et al., 2014). It is currently unknown whether these effects are limited to the right angular gyrus as most functions of the angular gyrus are represented bilaterally and hemispheric lateralization has been inconsistent across studies (compare Table 1; Seghier, 2013), potentially due to observed individual differences in hemispheric lateralization (Farrer et al., 2008).

Thus, the finding of Study 1 could be well interpreted within the framework of dissociation studies and the fact that the association can be detected bilaterally if the statistics are not corrected for whole-brain testing provides some additional reassurance.

Greater gray matter volume in the angular gyrus could then either be a structural diathesis for the experience of dissociation or a plastic adaptation to altered activation patterns during frequent dissociative processing. Due to the cross-sectional nature of our data this distinction cannot be made here.

However, we were not able to replicate this finding in a comparable, slightly larger patient sample (Study 2). No significant correlation between dissociation severity and volume of the left angular gyrus in the region of interest was established neither in the patient nor the control group. In addition, the group comparison in Study 2 did not result in a significant difference in angular gyrus volume. The explorative, whole brain analysis revealed that patients exhibited a significantly reduced volume bilaterally in the dorsolateral prefrontal gyrus and the thalamus as compared to controls. This group difference is likely related to differences in dissociation severity as it was rendered non-significant upon covariation of FDS scores. The dorsolateral prefrontal gyrus has been implicated in emotion regulation (Dorfel et al., 2014), and one previous study reported reduced volume as well as a significant association with dissociation severity in PTSD patients (Nardo et al., 2012), however, another could not replicate this (Daniels et al., 2016). Reductions in thalamus volume have previously been associated with dissociative symptoms in DPD patients (Daniels et al., 2015), but not PTSD patients (Nardo et al., 2012; Daniels et al., 2016).

There are some notable differences between the two data sets outlined below. However, whether these are significant enough to potentially explain the failed replication attempt remains unknown. While the two patient samples were both convenience samples from the same, specialized treatment center and their average target symptom scores were comparable (no significant group differences for dissociation severity and childhood maltreatment), the sample of Study 2 exhibited significantly higher depression scores (and convergently also received more comorbid diagnoses for major depression) than the sample of Study 1. However, covarying out the depression scores did not render the association between dissociation severity and regional gray matter volume significant.

In addition, no significant correlation between childhood trauma severity and dissociation scores was established in Study 2, while it was highly significant in Study 1. This is noteworthy as a large review found consistently significant associations between childhood trauma severity and dissociation scores across several samples of both healthy controls and patients for the dissociation instrument used in our study (Spitzer et al., 2015). There is no apparent reason as to why this might be different in the second sample as the dissociation measure is known to have a good reliability (re-test over the period of 2 weeks: healthy students *r* = 0.82; in-treatment patients *r* = 0.80; Spitzer et al., 2015). There were slight differences in assessment procedures, but they are unlikely to be associated with this finding: In Study 2, patients were asked to fill in the assessment instruments when they were admitted to in-patient treatment, i.e., likely before any in-depth discussion of their dissociation symptoms with a clinician might have happened. In contrast, patients from Study 1 were administered the questionnaires at a slightly later time point during their treatment, i.e., after having received on average approximately 4 weeks of intervention. To what extent dissociative symptoms had been discussed by then and if this could have impacted their symptom reporting is unknown. A recent study indicated that intense targeted activation can impact the volume of the angular gyrus very rapidly (Schmidt-Wilcke et al., 2018), thus behavioral differences between the two groups could in theory have had an impact. Specialized treatment approaches as the one delivered in the clinic where both study samples were recruited result in significant reductions of dissociative symptoms (reflected in a low re-test reliability for the assessment instrument, *r* = 0.53 in an in-patient sample; Spitzer et al., 2015). However, in this case a lower correlation between childhood trauma severity and dissociation scores would have been expected in Study 1, but not Study 2 (as this group had not received any treatment by the time they were assessed).

Potentially, the observed alterations could also be due to differential effects of psychotropic medication (Lanius et al., 2010). However, the angular gyrus specifically has never been shown to be affected by psychotropic medication (Lai and Wu, 2013; Samann et al., 2013; Talati et al., 2015) and it seems unlikely that potential medication effects would differ enough between the two samples as the participants exhibited a similar usage of psychotropic medication. Covarying the intake of psychotropic medication also did not render the results of the two studies more coherent.

On a more technical note, it might be worth mentioning that patients from Study 1 were scanned on a 1.5 Tesla scanner vs. a 3 Tesla scanner used in Study 2 and different reconstruction filter settings in the two scanners cannot be excluded.

In short, we noted several limitations to our studies: small samples sizes, different clinical characteristics in terms of depression severity and the association of dissociation severity with childhood trauma severity, different medication profiles in the two groups, and differences in the technical instrumentation. Both studies were limited to female participants and hence no conclusions can be drawn regarding potential effects in males.

However, it seems unlikely that the described differences in sample composition and data acquisition can fully account for the divergence of the results. It is possible that the selection of participants was biased in undetected ways, i.e., that not both samples are good representatives of the population of patients. Thus, it is possible that a true effect was detected in Study 1 but not Study 2. Vice versa, and taking into account that a severe publication bias in this field has previously been demonstrated convincingly (Fusar-Poli et al., 2014), it also seems possible that the results of Study 1 might be spurious. Both our studies, as most patient studies in this realm, suffer from small samples sizes. Small sample sizes, in turn, are associated with limited reliability (Button et al., 2013) and thus limited replicability. Pooling patient data across different clinical centers (multi-center studies) might be the most obvious solution to this problem in the future. However, technical upgrades – like in our case – and differences in assessment instruments and procedures complicate this endeavor (Focke et al., 2011). Future studies should thus aim to pool data across many different centers (Dansereau et al., 2017), so that these differences can reliably be co-varied out. Alternatively, the publication of many small-scale studies as the ones presented here will help to eventually identify true associations using meta-analytical statistics. This would, however, require an effort to replicate previously published studies.

## Author Contributions

JD and IC drafted the manuscript. All authors contributed to revising the manuscript. JD, PH, KD, and IC were involved in the data analysis. AS, JS, and KW were involved in recruitment and clinical assessment of patients. AS and FR were involved in MRI data acquisition.

## Conflict of Interest Statement

The authors declare that the research was conducted in the absence of any commercial or financial relationships that could be construed as a potential conflict of interest.

## Footnotes

1 http://www.fil.ion.ucl.ac.uk/spm

2 http://www.fil.ion.ucl.ac.uk/spm/ext

3 http://dbm.neuro.uni-jena.de/vbm

4 http://www.ibm.com/analytics/us/en/technology/spss
