# Supplementary material for: Gray Matter Alterations Associated With Dissociation in Female Survivors of Childhood Trauma
Source: Front Psychol. 2019 Apr 5;10:738. doi: 10.3389/fpsyg.2019.00738 (PMC6460891; doi:10.3389/fpsyg.2019.00738)
Supplement: Supplementary file 1 [file Data_Sheet_1.PDF]

Supplementary material

			Childhood Trauma							Diagnosis of mental disorder								Medication					
ID	Age	BDI	CTQ sumscore	CTQ emotional abuse	CTQ physical abuse	CTQ sexual abuse	CTQ emotional neglect	CTQ physical neglect	Dissociation severity	Anxiety disorders (F40/41)	PTSD (F43.1)	Major depression (F32/33)	Personality disorders (F60/61)	Somatoform disorder (F45)	Substance abuse (F1)	Other disorders		Antidepressiva	Neuroleptica	Tranquilizer	Anticonsulvants	Analgetics	Other medication
Study 1																							
S1_10	40	18	16.6	24	21	5	22	11	37.4	□		□	□			□			□				
S1_13	50	20	16.0	14	17	17	19	13	13.9	□	□			□		□		□					□
S1_15	30	20	18.4	23	17	12	24	16	9.3			□	□		□	□							□
S1_20	40	33	15.6	20	21	5	21	11	27.7	□	□		□		□	□							□
S1_22	40	20	13.8	16	11	6	22	14	9.1							□							□
S1_24	20	17	11.4	14	10	6	20	7	8.9							□							□
S1_25	40	21	19.8	25	20	14	21	19	13.2	□	□	□		□				□					
S1_27	50	39	15.2	14	12	9	25	16	29.8							□		□	□				
S1_5	40	25	13.6	24	6	9	12	17	7.3							□		□					□
S1_8	30	10	12.8	19	10	12	11	12	9.8		□	□	□	□		□		□	□			□	
S1_9	50	8	13.0	17	11	12	13	12	39.5		□			□		□			□	□			
S2_21	60	19	11.2	9	14	11	9	13	3.2		□	□			□						□		
S2_23	50	35	19.2	19	22	25	14	16	52.7		□	□		□		□		□				□	□
S2_28	30	51	16.0	25	10	9	25	11	21.8		□	□		□				□		□	□		
S2_30	50	23	16.6	23	16	10	24	10	29.8		□	□	□					□	□	□			
S2_31	20	36	11.8	17	9	18	8	7	36.6		□	□		□	□	□						□	
S2_32	20	32	14.2	20	10	14	18	9	28.9			□	□					□					
S2_34	40	36	20.4	24	17	25	20	16	61.4		□	□		□		□		□	□			□	
S2_36	50	25	11.4	10	5	9	20	13	6.1		□	□				□							
S2_39	20	34	17.6	22	8	24	19	15	36.8		□		□			□		□					
S2_40	50	20	22.4	22	24	25	25	16	35.0		□	□		□		□							
S2_41	40	40	14.6	14	8	11	25	15	26.1			□											

**Mean**	39.6	26.5	15.5	18.9	13.6	13.1	19.0	13.1	24.7															
**SD**	10.1	10.4	3.1	4.8	5.4	6.4	5.3	3.1	15.5															
**N**										4	14	14	7	9	4	13		14	7	2	5	4	8	
Study 2																								
T1	20	44	20						79.0	□	□	□						□					□	
T2	20	40	13						25.0	□	□	□						□		□				
T3	30	34	11.6						39.5	□	□	□							□		□			
T4	30	35	15.2						30.5		□	□		□				□					□	
T5	30	30	14.4						53.0	□	□	□		□				□					□	
T6	30	50	22						70		□		□							□				
T7	30	44	14						43	□	□	□		□				□						
T8	30	44	14						43	□	□	□						□					□	
T9	30	23	17.2						33.5	□	□	□							□	□				
T10	40	62	12.8						11.0		□	□						□					□	
T11	40	36	21						18.5	□	□	□		□				□						
T12	40	38	23.2						45.0	□		□				□		□					□	
T13	40	28	19						5.5		□	□						□		□				
T14	40	41	21.6						34.5	□	□	□				□		□					□	
T15	50	49	17						23.0	□	□	□						□				□		
T16	50	47	14.6						24.5	□	□	□		□				□				□		
T17	50	27	18.8						8.0		□	□												
T18	50	40	16						20.5		□	□						□					□	
T19	50	36	11.4						29.5	□	□	□												
T20	50	25	21.6						27.5	□	□	□		□				□		□				
T21	50	42	24.6						20.5	□	□	□		□				□					□	
T22	50	56	19.8						69.5	□		□				□				□	□			
T23	50	45	22.2						21.5		□	□		□				□					□	
T24	60	25	20.8						19.0	□	□		□					□						
T25	60	34	16.6						7.0		□	□						□					□	
T26	60	45	13.4						20.5	□	□	□						□						
**Mean**	41.4	39.2	17.5	14.9	11.0	11.6	16.6	11.4	24.5															
**SD**	9.9	9.4	3.8	7.0	6.5	7.4	5.9	5.2	18.7															
**N**										18	24 9	24	2	8	13 0	3		20 58	2	6	2	2 9	11	8
C1	20	1	5.0						0.0															
C2	30	4	6.4						13.0															
C3	60	2	6.8						0.0															
C4	30	5	6.0						0.0															
C5	50	10	6.2						14.0															
C6	30	14	7.2						2.0															
C7	50	7	6.2						0.5															
C8	40	0	9.2						4.5															
C9	50	1	5.4						0.5															

C10	20	8	7.0						36.5															
C11	70	4	5.8						4.0															
C12	50	8	7.4						0.0															
C13	30	4	5.2						13.0															
C14	50	4	5.4						2.0															
C15	50	6	5.4						1.5															
C16	50	2	5.6						2.5															
C17	60	0	0.0						0.0															
C18	30	0	0.0						2.5															
C19	50	0	0.0						1.5															
C20	50	0	0.0						1.5															
C21	50	0	0.0						1.0															
C22	20	0	0.0						20.0															
C23	20	0	6.0						1.5															
C24	20	3	0.0						11.5															
C25	30	2	5.6						10.5															
**Mean**	39.7	3.4	4.5																					
**SD**	13.9	3.7	3.0																					

Table S1: Descriptive statistics of the sample.

SD = Standard deviation, N = Sample Size

For reasons of anonymity, the participants age has been downsampled to categories: 20= 18 to 24 years; 30= 25 to 34 years; 40= 35 to 44 years; 50=45 to 54 years; 60= 65 to 64 years.

In Study 2, T1 - T26 refers to the patient sample, C1 - C25 refers to the healthy controls.

MNI coordinates			T Score	Cluster Size k	Brain Region
**Positive correlation with dissociation severity, covaried for the severity of childhood traumatization**					
-44	-54	23	5.32	126	**Left angular gyrus**
**Positive correlation with dissociation severity, covaried for medication use with 5 nuisance regressors**					
-45	-56	24	6.06	56	**Left angular gyrus**
-58	-59	10	5.34	53	Left middle temporal gyrus
-41	-76	25	5.29	43	Left middle occipital gyrus
-39	42	3	4.57	31	Left inferior frontal gyrus (pars triangularis)
**Positive correlation with dissociation severity, covaried for depression scores [BDI]**					
-45	-56	24	5.4	172	**Left angular gyrus**

Table S2: **Results of Study 1 (n = 22) - grey matter volume in relation to dissociation severity under inclusion of different covariates.** Dissociation severity is operationalized with FDS scores (Fragebogen für Dissoziative Symptome).
All analyses were thresholded at $p < .001$ with a cluster extend of $k > 20$.
No significant negative correlations were detected.

MNI coordinates			T Score	Cluster Size k	Brain Region
**Positive correlation with dissociation severity**					
-42	50	0	4.36	59	Left middle Frontal Gyrus
-48	-63	-2	4.55	51	Left middle Temporal Gyrus
-38	-36	56	4.34	39	Left postcentral Gyrus
6	-45	74	5.20	29	Precuneus
**Negative correlation with dissociation severity**					
-24	-69	45	4.94	143	Left superior Parietal Lobule
**Positive correlation with dissociation severity, covaried for the severity of childhood traumatization**					
42	-65	3	4.4	69	Left middle temporal Gyrus
57	14	17	3.98	30	Right inferior frontal gyrus (pars opercularis)
-48	9	6	3.94	27	Left inferior frontal gyrus (pars opercularis)
-48	-65	0	3.94	27	Left middle temporal gyrus
6	-45	74	5.06	26	Precuneus
-45	50	-5	3.73	24	Left middle orbital gyrus
**Negative correlation with dissociation severity, covaried for the effect of childhood traumatization**					
-27	-63	48	3.88	20	Left superior parietal lobule
**Positive correlation with dissociation severity, covaried for medication use with 5 nuisance regressors**					
6	-45	74	5.32	34	Precuneus
-48	-67	2	4.6	58	Left middle temporal gyrus
-42	50	0	4.49	74	Left middle frontal gyrus
-38	-36	56	4.12	24	Left postcentral gyrus
**Negative correlation with dissociation severity, covaried for medication use with 5 nuisance regressors**					
-26	-68	44	3.93	151	Left inferior parietal lobule
**Positive correlation with dissociation severity, covaried for depression scores [BDI]**					
-35	54	-2	4.08	68	Left middle orbital gyrus

42	-65	2	4.33	61	Middle temporal gyrus
-48	-65	0	4.6	52	Middle temporal gyrus
57	14	17	3.99	41	Middle temporal gyrus
6	-45	74	5.06	25	Precuneus
-48	9	6	3.89	21	Inferior frontal gyrus
**Negative correlation with dissociation severity, covaried for depression scores [BDI]**					
-36	18	30	3.9	22	Inferior frontal gyrus (pars triangularis)

Table S3: **Results of Study 2 (n = 26) - grey matter volume in relation to dissociation severity and under inclusion of different covariates.** Dissociation severity is operationalized with FDS scores (Fragebogen für Dissoziative Symptome).
All analyses were thresholded at $p < .001$ with a cluster extend of $k > 20$.
